# Psychological Burden and Coping Strategies Among Caregivers of Patients With End-Stage Cancer

**DOI:** 10.7759/cureus.108821

**Published:** 2026-05-13

**Authors:** Theodora Mamaki, Antonia Kalogianni, Maria Polikandrioti, Niki Pavlatou, Evgenia Stergiopoulou, Georgia Toulia

**Affiliations:** 1 Department of Nursing, University of West Attica, Athens, GRC

**Keywords:** cancer, caregivers, coping strategies, patients, psychological burden

## Abstract

Purpose: The present cross-sectional study investigated psychological burden and coping strategies among informal caregivers of patients with end-stage cancer, with emphasis on anxiety, depression, stress, and sociodemographic and caregiving-related factors associated with caregiver burden.

Methods: The sample consisted of 300 informal caregivers of patients with end-stage cancer who were enrolled between 2023 and 2025 and completed the study questionnaires at a single time point. Caregiver burden was assessed with the Zarit Burden Interview (ZBI), psychological distress with the Depression Anxiety Stress Scales (DASS-21), and coping strategies with the Ways of Coping Questionnaire. Statistical analysis included descriptive statistics and multiple linear regression analysis, with the ZBI total score as the dependent variable. A p-value of <0.05 was considered statistically significant.

Results: Most caregivers experienced high psychological burden, with 292 participants (97.3%) classified in the high-burden category. Anxiety was particularly elevated, with 184 caregivers (61.3%) classified as having extremely severe anxiety, and emerged as a strong positive predictor of caregiver burden (B = 0.775, p < 0.001). The final regression model showed high predictive power (R² = 0.750, F(10, 289) = 86.638, p < 0.001). Coping strategies such as positive reappraisal (B = -5.284, p < 0.001), problem solving (B = -5.371, p < 0.001), and social support (B = -2.431, p < 0.001) were associated with lower burden.

Conclusions: The findings indicate substantial psychological burden among informal caregivers of patients with end-stage cancer, with anxiety playing a central predictive role. Adaptive coping strategies were associated with lower burden, highlighting the need for systematic psychosocial assessment and targeted caregiver-support interventions within palliative care services.

## Introduction

Caregivers of patients with end-stage cancer constitute a critical, yet often overlooked, component of the healthcare system, playing a key role in providing physical, emotional, and psychosocial support to patients. End-of-life care is characterized by increased demands, including management of severe symptoms, support with existential issues, and constant confrontation with loss and death [1]. In recent decades, the gradual shift of oncology care from hospital settings to home-based care has substantially increased the responsibilities placed on families and informal caregivers, consequently heightening their exposure to psychological distress [2,3].

International literature consistently demonstrates that caregivers of cancer patients experience high levels of anxiety, depression, and stress, often exceeding those observed in the general population and approaching levels reported in clinical populations [4,5]. The emotional demands associated with caregiving, combined with restrictions in social life, financial strain, and physical fatigue, can negatively affect caregivers' quality of life and increase the risk of mental health problems [6].

Among the various dimensions of psychological burden, anxiety appears to play a particularly prominent role among caregivers of patients with end-stage disease. Uncertainty about the course of the disease, fear of deterioration, constant vigilance, and a sense of inadequacy in managing critical situations may contribute to persistently high levels of anxiety symptoms [7,8]. At the same time, caregiver depression has been associated with prolonged exposure to stressful circumstances, loss of personal roles, and sustained emotional exhaustion [9].

Caregiver burden is a multidimensional phenomenon shaped by the interaction of individual, social, and caregiving factors. Characteristics of caregivers, such as gender, age, relationship with the patient, and cohabitation status, have been associated with variations in perceived burden [10]. Furthermore, the strategies caregivers use to cope with caregiving demands play a decisive role in shaping psychological well-being.

Coping strategies such as positive reappraisal, problem solving, and seeking social support have been associated with lower levels of psychological burden, while more passive or avoidant strategies have been associated with increased anxiety and depression. Despite the growing body of international research on caregiver burden, relatively few studies in Greece have systematically investigated caregiver burden in end-stage cancer and the role of coping strategies in this context.

Despite the growing body of international research on caregiver burden, relatively few studies in Greece have systematically investigated psychological burden among informal caregivers of patients with end-stage cancer and the role of coping strategies in this context. Within this framework, the present cross-sectional study aimed to investigate levels of depression, anxiety, and stress among caregivers of patients with end-stage cancer, assess the degree of caregiver burden, and examine coping strategies used by caregivers. In addition, the study aimed to identify psychological, coping-related, sociodemographic, and caregiving-related factors associated with caregiver burden. By addressing these aims, the study seeks to contribute to a more comprehensive understanding of factors that may exacerbate or alleviate psychological burden among caregivers and inform interventions aimed at improving caregiver well-being and the quality of care provided to patients.

## Materials and methods

Study design and participants

This was a cross-sectional observational study using convenience sampling. Participants were enrolled between 2023 and 2025 from an oncology clinic. The extended period refers to participant enrollment; each caregiver completed the study questionnaires once, at a single time point.

The sample consisted of 300 informal caregivers of patients with end-stage cancer. Eligible participants were adult informal caregivers, such as spouses, parents, siblings, or other close relatives, who had an active role in the daily or systematic care of a patient with a terminal cancer diagnosis, regardless of whether they lived with the patient. Caregivers were included if they were aged 18 years or older, were able to understand and complete the questionnaires, and provided informed consent. Caregivers who were unable to complete the questionnaires because of severe cognitive or communication difficulties were excluded. Women accounted for 200 participants (66.7%), while men accounted for 100 participants (33.3%). The mean age of participants was 51.9 years (SD = 13.7), ranging from 28 to 87 years.

Ethical considerations

The study was conducted in accordance with the principles of the Declaration of Helsinki and was approved by the Ethics Committee of the University of West Attica, Athens, Greece, approval number 56969/13-06-2023, dated: 04/07/2023. All participants were informed about the purpose of the study, the voluntary nature of participation, confidentiality of responses, and their right to withdraw at any time without any consequences. Written informed consent was obtained from all participants before questionnaire completion.

Measurement instruments

Caregiver burden was assessed using the Zarit Burden Interview (ZBI) [11], a widely used instrument in clinical and research settings. The ZBI was originally developed by Zarit et al. [11]. It consists of 22 items rated on a five-point Likert scale ranging from 0 (“never”) to 4 (“nearly always”), with total scores ranging from 0 to 88; higher scores indicate greater caregiver burden. The validated Greek version of the ZBI was used [12]. The Greek validation study reported satisfactory psychometric properties, including a four-factor structure, adequate internal consistency, construct validity through significant correlations with the Bakas Caregiving Outcomes Scale, and known-groups validity [12]. In the present study, the ZBI demonstrated high internal consistency (Cronbach’s alpha = 0.815).

Psychological distress was assessed using the Depression Anxiety Stress Scales (DASS-21) [13]. The DASS was originally developed by Lovibond and Lovibond [13], and the validated Greek version was employed [14]. The Greek validation study used forward and backward translation procedures and supported the reliability and validity of the instrument, reporting high internal consistency for the total DASS and subscales, significant correlations with established anxiety and depression measures, and a three-factor structure consistent with the depression, anxiety, and stress dimensions [14]. The DASS-21 consists of 21 self-report items divided into three subscales measuring depression, anxiety, and stress. Each item is rated on a four-point Likert scale from 0 to 3. In the present study, internal consistency was satisfactory for the depression subscale (Cronbach’s alpha = 0.799), while the anxiety (Cronbach’s alpha = 0.678) and stress (Cronbach’s alpha = 0.650) subscales demonstrated acceptable reliability for research purposes.

Coping strategies were assessed using the Ways of Coping Questionnaire, which was developed within the Lazarus and Folkman stress-and-coping framework and further described in the revised coping literature [15]. The Greek adaptation by Karadimas was used [16]. The instrument consists of 38 items grouped into eight subscales representing different cognitive, emotional, and behavioral coping strategies. Responses are rated on a four-point Likert scale from 0 to 3, indicating the frequency of use of each strategy. In the present study, internal consistency across subscales ranged from acceptable to high (Cronbach’s alpha = 0.658-0.810). In addition to the standardized scales, a structured questionnaire was used to record sociodemographic characteristics and caregiving-related data.

## Results

Statistical analysis

Statistical analysis was conducted using SPSS version 22.0 (IBM Corp., Armonk, NY, USA). Descriptive statistics were used to summarize sample characteristics and study variables. Categorical variables are presented as N (%), and continuous variables are presented as mean ± standard deviation (SD), unless otherwise stated.

Multiple linear regression analysis was performed to examine factors associated with caregiver psychological burden. The dependent variable was the total ZBI score. Candidate independent variables included DASS-21 depression, anxiety, and stress scores; coping strategies assessed by the Ways of Coping Questionnaire (positive reappraisal, problem solving, social support, wishful thinking, seeking divine help, passive acceptance, emotional avoidance, and assertive problem solving); living with the patient; availability of caregiving assistance; and caregiver relationship to the patient, with parent used as the reference category. Variable selection was conducted using the backward method. Model assumptions, including multicollinearity, independence of errors, normality of residuals, and homoscedasticity, were assessed. All statistical tests were two-tailed, and a p value of <0.05 was considered statistically significant.

Findings

The distribution of caregivers across severity levels of depression, anxiety, and stress is presented in Table 1. For depression, 44 participants (14.7%) were classified within the normal range, 52 (17.3%) reported mild symptoms, 85 (28.3%) moderate symptoms, 76 (25.3%) severe symptoms, and 43 (14.3%) extremely severe symptoms. Anxiety levels were markedly elevated, with only 12 caregivers (4.0%) classified within the normal range and 13 (4.3%) within the mild category. A total of 41 caregivers (13.7%) reported moderate anxiety, 50 (16.7%) severe anxiety, and 184 (61.3%) extremely severe anxiety. Regarding stress, 77 caregivers (25.7%) were classified as normal, 84 (28.0%) as mild, 94 (31.3%) as moderate, and 45 (15.0%) as severe, while no participants were classified in the extremely severe stress category. 

**Table 1:** Evaluation of Caregivers According to Depression, Anxiety, and Stress Scales (DASS-21). Data are presented as N (%). Note: DASS-21 = Depression Anxiety Stress Scales-21. Percentages are calculated using the total sample size (N = 300).

DASS-21 subscale	Normal N (%)	Mild N (%)	Moderate N (%)	Severe N (%)	Extremely severe N (%)
Depression	44 (14.7%)	52 (17.3%)	85 (28.3%)	76 (25.3%)	43 (14.3%)
Anxiety	12 (4.0%)	13 (4.3%)	41 (13.7%)	50 (16.7%)	184 (61.3%)
Stress	77 (25.7%)	84 (28.0%)	94 (31.3%)	45 (15.0%)	0 (0.0%)

Table 2 illustrates the distribution of caregivers according to levels of burden. The vast majority of participants, 292 (97.3%), were classified as experiencing high caregiver burden, while only 8 (2.7%) participants fell into the low-burden category. 

**Table 2:** Caregiver Burden Scale. Data are presented as N (%). Note: Percentages are calculated using the total sample size (N = 300).

Burden level	N (%)
Low burden	8 (2.7%)
High burden	292 (97.3%)
Total	300 (100.0%)

Table 3 presents the mean values, standard deviations, and score ranges for the eight subscales of the Ways of Coping Questionnaire. Mean scores for positive reappraisal, problem solving, and social support were each 1.8. The mean score for wishful thinking was 1.6, while the mean scores for seeking divine help, passive acceptance, and emotional avoidance were each 1.5. Assertive problem solving showed the highest mean score, at 2.1, suggesting that this strategy was used most frequently by caregivers.

**Table 3:** Ways of Coping Questionnaire. Data are presented as mean ± SD and range. Note: SD = standard deviation. Scores range from 0 to 3, with higher scores indicating more frequent use of the coping strategy.

Subscale	Minimum	Maximum	Mean ± SD
Positive reappraisal	0.0	3.0	1.8 ± 0.7
Problem solving	0.3	3.0	1.8 ± 0.7
Social support	0.0	3.0	1.8 ± 0.6
Wishful thinking	0.0	3.0	1.6 ± 0.7
Seeking divine help	0.0	3.0	1.5 ± 0.8
Passive acceptance	0.0	3.0	1.5 ± 0.7
Emotional avoidance	0.0	3.0	1.5 ± 0.8
Assertive problem solving	0.0	3.0	2.1 ± 0.7

Table 4 presents the final multiple linear regression model predicting caregiver psychological burden. The total ZBI score was used as the dependent variable. Candidate predictors initially included depression, anxiety, stress, coping strategies, living with the patient, availability of caregiving assistance, and caregiver relationship to the patient. Variable selection was conducted using the backward method.

**Table 4:** Multiple Linear Regression Model Predicting Caregivers' Psychological Burden. Data are presented as B, standard error, Beta, t statistic, and p value. Note: B = unstandardized regression coefficient; Std. Error = standard error; Beta = standardized regression coefficient. A p value of <0.05 was considered statistically significant.

Model	Variable	B	Std. Error	Beta	t	p value
7	Constant	64.698	2.679		24.149	<0.001
	Anxiety	0.775	0.097	0.256	7.984	<0.001
	Positive reappraisal	-5.284	0.545	-0.325	-9.689	<0.001
	Problem solving	-5.371	0.482	-0.353	-11.137	<0.001
	Social support	-2.431	0.615	-0.140	-3.950	<0.001
	Wishful thinking	-4.026	0.542	-0.245	-7.424	<0.001
	Passive acceptance	-1.360	0.494	-0.089	-2.752	0.006
	Living with the patient	-3.263	0.882	-0.136	-3.699	<0.001
	Availability of caregiving assistance	3.490	0.757	0.154	4.611	<0.001
	Spouse vs. parent	-5.101	1.225	-0.165	-4.164	<0.001
	Sibling vs. parent	-3.625	0.974	-0.132	-3.720	<0.001

The final model demonstrated high predictive power, with an R² value of 0.750 and F(10, 289) = 86.638, p < 0.001. The model showed no evidence of autocorrelation, as indicated by the Durbin-Watson statistic (1.758, acceptable range 1-3), and no multicollinearity problems were observed (variance inflation factor (VIF) < 10). The assumptions of normality and homoscedasticity were met to a satisfactory degree.

In the final model, anxiety was positively associated with caregiver burden (B = 0.775, p < 0.001), indicating that higher anxiety scores were associated with higher ZBI scores. In contrast, several coping strategies were associated with lower burden, including positive reappraisal (B = -5.284, p < 0.001), problem solving (B = -5.371, p < 0.001), social support (B = -2.431, p < 0.001), wishful thinking (B = -4.026, p < 0.001), and passive acceptance (B = -1.360, p = 0.006). Living with the patient was also associated with lower burden scores (B = -3.263, p < 0.001). Compared with parents, spouses (B = -5.101, p < 0.001) and siblings (B = -3.625, p < 0.001) reported lower burden. Availability of caregiving assistance showed a positive association with caregiver burden (B = 3.490, p < 0.001), a finding that may reflect the fact that assistance is more often mobilized in situations where care needs and burden are already higher.

## Discussion

The present study showed that informal caregivers of patients with end-stage cancer experience substantial psychological burden, with anxiety as a particularly prominent component, while depression and stress were also common. This finding is consistent with international literature indicating that informal caregivers of oncology patients constitute a vulnerable group at increased psychosocial risk, burdened both emotionally and functionally [2,3,6].

A notable finding was the very high proportion of caregivers classified as having extremely severe anxiety. This result should be interpreted within the clinical context of end-stage cancer care. Caregivers in this setting often face uncertainty about disease progression, fear of sudden deterioration, anticipatory grief, continuous vigilance, and perceived responsibility for managing complex symptoms at home. In addition, the use of convenience sampling from the oncology clinic setting may have increased the likelihood of including caregivers with higher levels of distress. Therefore, although the finding highlights the importance of systematic caregiver assessment, it should not be overgeneralized to all caregivers of patients with cancer.

Anxiety emerged as a strong psychological predictor of caregiver burden. This finding is consistent with studies highlighting the central role of anxiety and stress-related processes in shaping the psychological state of caregivers, especially in end-stage disease, where uncertainty, anticipation of loss, and constant vigilance may intensify emotional burden [3,7,8]. At the same time, chronic exposure to stress has been associated with biological dysregulation mechanisms that may affect both physical and mental health [7,8].

The results of the study also showed that the majority of participants experienced high levels of burden, highlighting the care of patients with end-stage cancer as one of the most demanding forms of informal care. Increased burden has been associated with multiple factors, such as deterioration of the patient's condition, intensity of symptoms, and need for continuous care, findings that are consistent with previous studies [4,9,10]. In addition, the shift of care from hospital to home has significantly increased the burden placed on families and informal caregivers [1].

Regarding coping strategies, the findings indicate that active and adaptive strategies, such as positive reappraisal, problem solving, and seeking social support, were associated with lower levels of psychological burden. This result is consistent with literature recognizing the protective role of functional coping strategies against chronic stress [2,6]. In the final regression model, passive acceptance also showed a small negative association with burden. This finding should be interpreted cautiously and may reflect a form of contextual acceptance in end-stage illness rather than maladaptive avoidance.

Furthermore, the study suggests that psychological burden is not a unidimensional phenomenon but is influenced by psychological, social, and caregiving-related factors. Cohabitation with the patient, availability of support, and relationship with the patient appeared to shape the caregiving experience. These findings are consistent with recent qualitative approaches highlighting the complexity of caregiver experiences and the dynamic interaction between personal and environmental parameters [17]. Stress-related immune dysregulation has also been described as a possible pathway linking chronic stress with adverse health outcomes, further supporting the need for early recognition and management of caregiver distress [18].

Limitations

The present study has several limitations that should be considered when interpreting the findings. First, the cross-sectional design does not allow causal relationships between variables to be established; therefore, conclusions regarding the direction of influence among psychological, social, and caregiving-related factors cannot be drawn. Second, participants were enrolled using convenience sampling from an oncology clinic in Greece, which may limit representativeness and may have contributed to the high levels of anxiety and burden observed. Third, data collection was based on self-reported assessment tools, which may introduce social desirability bias or subjective assessment error. Fourth, although the sample size was adequate, the sample cannot be considered fully representative of all caregivers of patients with end-stage cancer, and generalization of the findings should be made with caution. Fifth, the study did not include some potentially important factors, such as duration of caregiving, severity of illness, functional status of the patient, and caregivers’ previous psychiatric history, which may influence burden. Finally, the complexity of end-of-life caregiving suggests that future research should complement quantitative findings with qualitative methods to understand caregivers’ experiences in greater depth.

## Conclusions

The findings of the present study indicate that informal caregivers of patients with end-stage cancer experience substantial psychological burden, with anxiety playing a central predictive role. Adaptive coping strategies, including positive reappraisal, problem solving, and social support, were associated with lower burden, highlighting their potential value as targets for caregiver-support interventions. Systematic psychosocial assessment and timely interventions within palliative care services are needed to identify caregivers at increased risk and to promote more effective coping, caregiver well-being, and quality of care for patients.
